# microRNA-449a modulates medullary thymic epithelial cell differentiation

**DOI:** 10.1038/s41598-017-16162-2

**Published:** 2017-11-21

**Authors:** Pengfei Chen, Haohao Zhang, Xiaohua Sun, Yiming Hu, Wenxia Jiang, Zhanjie Liu, Sanhong Liu, Xiaoren Zhang

**Affiliations:** 1 Department of traumatic orthopedics, Shenzhen Longhua District Central Hospital, Shenzhen, 518110 China; 2 Key Laboratory of Stem Cell Biology, Shanghai Institutes for Biological Sciences, University of Chinese Academy of Sciences, Chinese Academy of Sciences, Shanghai, 200031 China

## Abstract

Medullary thymic epithelial cells (mTECs) ectopically express a diversity of peripheral tissue-restricted antigens (PTAs) and provide unique cues for the expansion, maturation and selection of a repertoire of functionally diverse T lymphocytes. Genetic deletion of all mature microRNAs in thymic epithelial cells (TECs) results in premature thymic involution, progressive disorganisation of the thymic epithelium, and alteration in thymic T cell lineage commitment, consequently eliciting autoimmune disorders. In the present study, we identified that microRNA-449a (miR-449a), a member of miR-449 cluster, regulated mTEC differentiation. Expression of miR-449a was induced by RANK ligand in mouse fetal thymus. In *in vitro* studies, overexpression of miR-449a induced thymic epithelial progenitor cells (TEPCs) differentiation into mature mTECs. Despite abundant expression of miR-449a in developing thymus, miR-449a-mutant mice exhibited normal thymic development. This might be partially due to in miR-449a-mutant thymus the up-regulation of miR-34a which shared similar seed sequence with miR-449a. However, thymic expression of miR-449/34 sponge which was able to neutralize the function of miR-449/34 family members significantly reduced the number of mature Ly51^-^MHCII^hi^ mTECs. Taken together, our data suggested that miR-449a modulated mTEC differentiation, and members of miR-34 cluster functioned redundantly to rescue miR-449a deficiency in thymus development.

## Introduction

The thymus containing thymic epithelial cells (TECs) that form a complex three-dimensional meshwork structure provides the microenvironment to drive the differentiation of bone marrow-derived hematopoietic precursors to mature T lymphocytes^1^. TECs consist of cortical thymic epithelial cells (cTECs) and medullary thymic epithelial cells (mTECs) which form discrete intrathymic microenvironments, thymic cortex and thymic medulla respectively. Each is specialized for mediating a particular aspect of thymocytes development^2,3^. The bone marrow-derived progenitors go through a consecutive process including release from bone marrow niches into the blood^4,5^ and exit from the circulation to settle in the thymus at the cortico-medullary junction (CMJ)^6,7^. On entering the thymus, these early thymic progenitors (ETP) undergo an ordered process of development from CD4/CD8 double negative stage (CD4^−^/CD8^−^) to double positive stage (CD4^+^/CD8^+^) during their migration from CMJ through the cortex to the outer subcapsular zone (SCZ) and then back to the cortex^8–10^. During this process, progenitors become committed to the αβ or γδT cell lineage^11,12^ at DN3 stage and undergo β-selection^13^; the resulting immature single positive (ISP) intermediate cells then differentiate into double positive cells and undergo positive selection by recognization of self-peptide/MHC complexes expressed on cTECs^14,15^. Positively selected thymocytes then enter the medulla, where T cells express T cell receptors with high affinity for self-antigens are clonally depleted by apoptosis^16–18^.

The crucial role of mTEC in establishing T cell central tolerance is attributed to the expression and presentation of a diversity of peripheral tissue-restricted antigens^17,19–21^. Recent studies have elucidated a battery of regulators underlying the differentiation and function of mTEC, among which the identification of autoimmune regulator (Aire) was a breakthrough in the study of mTEC biology^19^. Members of the tumor necrosis factor receptor (TNFR) family and their downstream canonical/alternative NF-κB pathways are involved in the differentiation and function of mTECs. Mice deficient with RANK^22,23^, CD40^23^, lymphotoxin β receptor^24,25^, NF-κB inducing kinase (NIK)^26^, IκB kinase (IKK) α^27^, RelB^28^, and Traf6^29^ exhibited variable defects in mTEC.

MicroRNAs (miRNAs) are a class of small (19~22nt), noncoding RNAs that mediate sequence-dependent post-transcriptional gene repression by translational inhibition and/or mRNA destabilization. Approximately over 10,000 miRNAs have been identified to exert their effects in normal organism development and pathogenesis^30–32^. However, the molecular mechanisms of miRNAs underlying thymus development remain less understood. Adrian Liston’s group reported the function of thymic epithelial miRNA network in infection-associated thymic involution by *Foxn1* mediated conditional knockout of *Dicer* in mouse model^33^. The deletion of *Dicer* and therefore all mature miRNAs in thymic epithelial cells result in premature thymic involution, progressive disorganisation of the thymic epithelium, alteration in thymic T cell lineage commitment, and consequently elicit autoimmune disorders^33,34^. In accordance with these discoveries, *Foxn1* mediated conditional knockout of *DGCR8*, a gene whose product is responsible for canonical cleavage of miRNAs, results in severe loss of Aire^+^ mTECs and breaks down the negative selection in thymus^35^. A transcriptome analysis of murine thymic epithelial cells reveals miRNA expression profiling that is closely correlated with age-related thymic atrophy, indicating the function of miRNAs in control of thymic aging process^36^. Thus, miRNAs are reasonably playing crucial roles in thymic epithelial cell differentiation and function.

We identified miR-449a in 2-DG FTOC (2-DG FTOC, 2′-deoxyguanosine (2-DG) treated Fetal Thymus Organ Culture) treated with recombinant human RANK ligand. Expression of miR-449a was induced by RANK ligand in fetal thymus and overexpression of miR-449a could induce TEPC differentiation *in vitro*. Neutralization of miR-449a and other miR-449/34 family members reduced the number of mature MHCII^hi^ mTECs in thymus. Our results revealed a new function of miR-449a in regulation of mTEC differentiation.

## Materials and Methods

### Animals

The miR-449a mutant mice (miR-449a^Ins/Ins^ and miR-449a^Del/Del^) were generated by Shanghai Bioray Biotech Co., Ltd. All animals were housed and maintained in specific-pathogen-free conditions. All animal experiments were performed in compliance with the guide for the care and use of laboratory animals and were approved by the institutional biomedical research ethics committee of the Shanghai Institutes for Biological Sciences, Chinese Academy of Sciences.

Primers used for genotyping:

F1: CACAATTCTATCTCTAGGCC

F2: GCTGGTTGAGTATGTGAG

R: GGGCAAATACACAAGGC

F1 and R primer pair for miR-449a^Ins/Ins^ genotyping, a 336 bp band indicates insert mutation, and further sequencing is needed to distinguish heterozygous and homozygous mutation. F2 and R primer pair for miR-449a^Del/Del^ genotyping, a 314 bp band indicates wild type or heterozygous mutation, homozygous mutation results in no amplification.

### Cell lines and cell culture

TSC (thymic epithelial progenitor cell line) cells were established in our lab previously^37^. TSC cells were treated with 100 ng/ml recombinant human RANKL (R&D, 390-TN-010) for 2 or 4 days.

To stably overexpress miR-449a in TSC cells, TSC cells were infected with empty control lentivirus or lentivirus expressing miR-449a and selected with 1ug/ml puromycin for 4 days. The stable cell lines were thereafter named as TSC Control or TSC miR-449a. Overexpression of miR-449a (>300 fold) was then confirmed by q-PCR.

### Fetal Thymus Organ Culture, FTOC

Thymic lobes were isolated from E14.5 embryos and cultured for 4 days on nucleopore filters (Whatmann, NJ) placed in RPMI1640 (Invitrogen), supplemented with 10% fetal bovine serum (Invitrogen), 2 mM L-glutamine (Invitrogen), 50 µM 2-mercaptoethanol (Sigma-Aldrich) and 1.35 mM 2′-deoxyguanosine (2-DG, Sigma-Aldrich), as previously described^23^. Fetal thymic lobes (2-DG FTOC) were cultured in RPMI1640-10%FBS medium and infected with lentivirus carrying miR-449a or miR-449/34 sponge expressing vectors. For *in vitro* differentiation, fetal thymic lobes (2-DG FTOC) were cultured with 100ng/ml recombinant human RANKL (R&D, 390-TN-010) for 4 days.

### Plasmids construction

MiR-449a was amplified from mouse genome using forward primer: 5'-TGA ATT CAC TTA GCC TCA GCC ACT C-3' and reverse primer: 5'-TGT CTA GAT AAT GTC AAG CTA GGA C-3' and cloned into plvx-IRES-EGFP (Clontech).

To clone miR-449/34 sponge, forward sequence: 5'-gatccACCAGCTAACTATCACTGCC ACGATACCAGCTAACTATCACTGCCAACGCGACCAGCTAACTATCACTGCCACGATACCAGCTAACTATCACTGCCAACG CGACCAGCTAACTATCACTGCCACGATACCAGCTAACTATCACTGCCAttttttg-3' and reverse sequence: 5'-aattcAAAAAATGGCAGTGATAGTTAGCTGGTATCGTGGCAGTGATAGTTAGCTGGTCGCGTTGGCA GTGATAGTTAGCTGGTATCGTGGCAGTGATAGTTAGCTGGTCGCGTTGGCAGTGATAGTTAGCTGGTATCGTGGCAGTGA TAGTTAGCTGGT g-3' were synthesized, annealed and cloned into plvx-shRNA2 (Clontech).

### Western blot

The cells were harvested and washed with cold phosphate buffer solution (PBS) once. Add 100 µl lysis/loading buffer (0.25 M Tris-HCl, 10% SDS, 0.5% Bromophenol blue, 50% Glycerinum, 7.71% Dithiothreitol), boiling for 10 minutes.

For each sample, 10-20 μl of protein lysate was separated by SDS-PAGE, transferred electrophoretically to a PVDF membrane (Immobilon P, Millipore) and immunoblotted with primary and peroxidase-conjugated secondary antibodies in 5% non-fatty milk. Detection of the bound antibody was performed by SuperSignal west pico Chemiluminescent Substrate (Pierce). Antibodies to Aire (N-20), GAPDH (G-9), SATB2 (SATBA4B10), RelB (A-9) and NF-κB p52 (K-27) were purchased from Santa Cruz Biotech Inc. Monoclonal antibody to DNMT3a was generated in Dr. Guoliang Xu’s lab (Institute of Biochemistry and Cell Biology, SIBS, CAS).

### Thymic *in situ* injection

Virus expressing Control-GFP or miR-449/34 sponge-GFP were packaged according to Lenti-X™ shRNA Expression Systems User Manual (Clontech). Control-GFP virus and miR-449/34 sponge-GFP virus were concentrated by ultracentrifugation and stored at −80 °C. The GFP protein acted as a reporter gene of miR-449/34 sponge expression.

3-week old C57BL/6 mice were narcotized, injected with about 30 μl of virus per lobe of thymus at the interstice between the second and third rib. Injection was conducted once every other day for 3 times. 2 days after the last injection, the thymus were harvested for analysis.

### Flow cytometry

The thymi were harvested, washed with cold PBS twice, minced and digested with Liberase TH (Roche) and DNase I (Roche). Thymic cells were stained with antibodies: EpCAM (G8.8, Biolegend, 118204), Ly51 (Biolegend, 108312), MHCII (BD sciences, 562366), CD45 (eBioscience, 45-0451-82) for 30 min on ice. mTECs were gated as CD45^−^EpCAM^+^Ly51^−^MHCII^+^.

For Aire staining, cells were stained with Foxp3/Transcription Factor Staining Buffer Set (eBioscience) and stained with Anti-Aire antibody (eBioscience, 51-5934-82) for 30 min on ice.

### Immunofluorescence

Frozen thymuses embedded in OCT compound were sliced into 8 µm-thick sections. Frozen sections were fixed with cold acetone for less than 5 minutes and stained with the following antibodies: rabbit polyclonal antibodies to Alexa-488 K5 (Covance), Alexa-647 K8 (Troma-1, Developmental Studies Hybridoma Bank), FITC-EpCAM (G8.8, Developmental Studies Hybridoma Bank), K14 (Covance) and Aire (M-300, Santa Cruz Biotech Inc.) followed by Alexa Fluor 568-conjugated anti-rabbit IgG antibody (Molecular Probes). Images were analyzed with TSC SP2 confocal laser-scanning microscope.

### RNA Extraction, RT-PCR and Q-PCR

RNA was isolated from cell lines or thymus samples using TRIzol Reagent (Invitrogen) and reverse transcripted using Transcript First Strand Synthesis Supermix (TransGen Biotech) according to the manufacturer’s instructions. Reverse transcription polymerase chain reactions (RT-PCR) were conducted using 2X Taq PCR MasterMix (TianGen Biotech).

All quantitative PCR (q-PCR) were performed using a 7500 Fast Real-Time PCR System (Applied Biosystems) in SYBR Premix Ex Taq reaction system (TaKaRa). Each sample was analyzed in triple replication. Relative quantification (RQ) was derived from the difference in cycle threshold (Ct) between the target gene and tubulin (ΔCt) as compared to control cell lines using the equation RQ = 2^−ΔΔCt^. Error bars represent standard deviation (SD), and statistical significance was calculated using a one-tailed, unpaired t-test. Relative mRNA or miRNA expression was summarized using mean ± SEM. All these results were calculated using student *t*-tests. p < 0.05 was considered to be significant. All the primers are listed in Table S2.

## Results

### Expression of miR-449a was induced by RANK ligand

**Figure 1:**
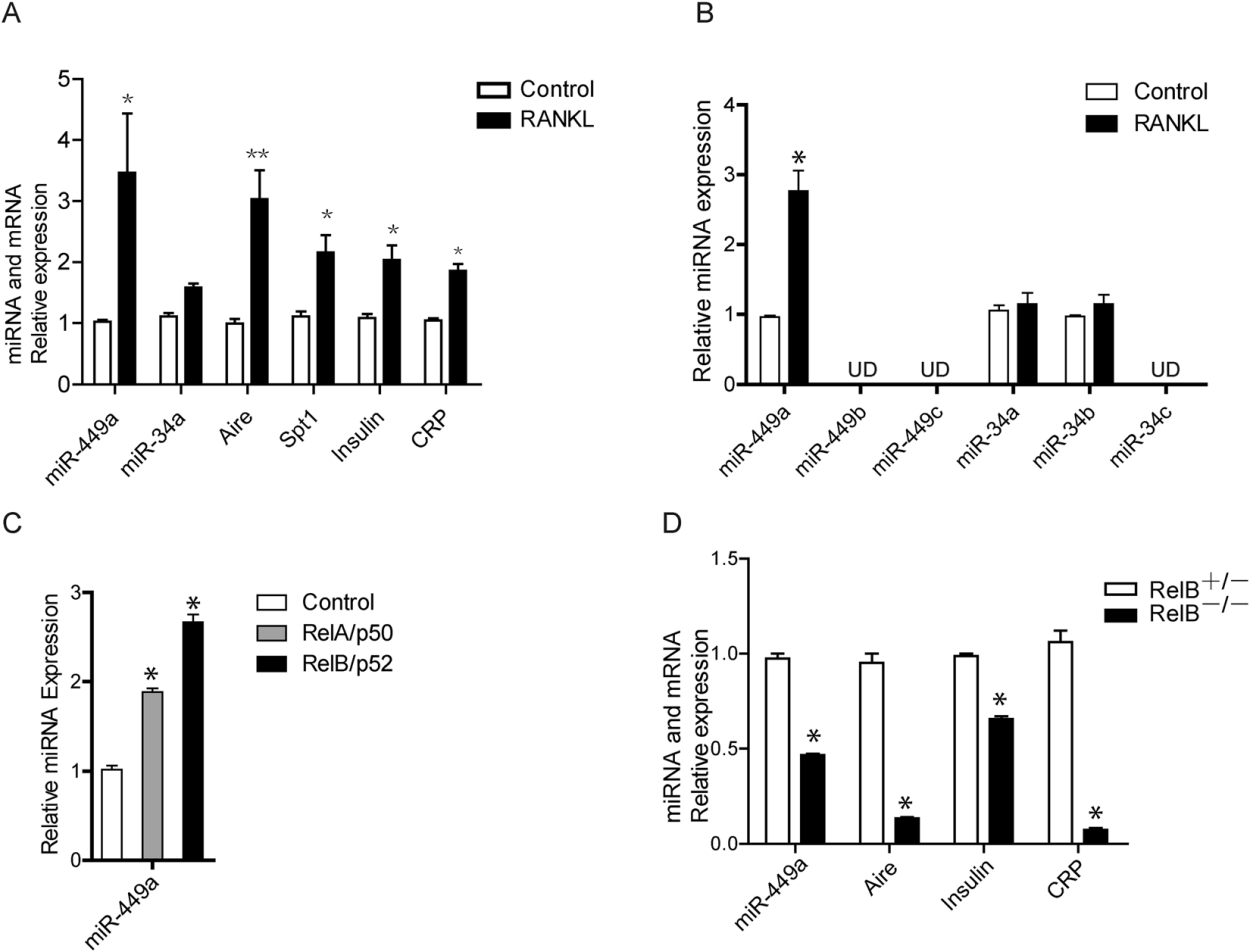
Expression of miR-449a was induced by RANKL. (**A**) q-PCR analyzed the expression of *miR-449a, miR-34a, aire, spt1, Insulin* and *CRP* in 2-DG FTOC treated with 100ng/ml recombinant human RANKL for 4 days. (**B**) q-PCR analyzed the expression of indicated genes in TSC cells treated with 100 ng/ml recombinant human RANKL for 2 days. (UD, undetectable). (**C**) q-PCR analysis of *miR-449a* expression in TSC cells overexpressing RelA/p50 or RelB/p52. (**D**) q-PCR analyzed the expression of indicated genes in *RelB*-deficient thymic epithelial cells. Bar graphs show means ± standard errors of at least three independent measurements. (*p < 0.05, **p < 0.01, n = 3).

RANK and its downstream NF-κB signaling were previously demonstrated to regulate the differentiation and function of mTEC^23^. To see if downstream miRNAs of RANK signaling regulate thymus development, we treated fetal thymi with RANK ligand (RANKL) for 4 days in 2-DG FTOC system. By RANKL stimulation, miR-449a was dramatically induced in 2-DG FTOC (Fig. 1A). And consistent with previous report, the expression of Aire as well as Aire-dependent PTAs (Spt1 and Insulin) and Aire-independent PTAs (CRP) were also significantly up-regulated (Fig. 1A).

We have previously established thymic epithelial progenitor cells (named as TSC) as an *in vitro* experimental system to study mTEC differentiation^37^. Stimulation with recombinant human RANKL induced the expression of Aire and Aire-dependent PTAs in TSC cells^37^. To see if expression of miR-449a could be induced by RANKL, TSC cells were treated with 100ng/ml RANKL for 2 days. Upon RANKL stimulation, expression of miR-449a was significantly increased while other members of miR-449 cluster were undetectable (Fig. 1B). MiR-449 cluster members (miR-449c/449b/449a) share similar seed sequence with miR-34 cluster members (miR-34a, miR-34b/34c) and constitute a conserved miRNA family^38–40^. Unlike miR-449a, expression of miR-34a, miR-34b and miR-34c remained unchanged or undetectable (Fig. 1B). In addition, overexpression of RelA/p50 or RelB/p52 in TSC cells induced miR-449a expression (Fig. 1C), indicating that RANKL and downstream canonical or non-canonical NF-κB activation could induce miR-449a expression. In contrast, *RelB*-deficient thymic epithelial cells showed decreased miR-449a expression as well as Aire and PTAs expression, which were consistent with previous report (Fig. 1D)^28^. These results demonstrated that RANKL and downstream canonical or non-canonical NF-κB activation were sufficient to induce miR-449a expression in TECs.

### MiR-449a expression profiling during thymus development

**Figure 2:**
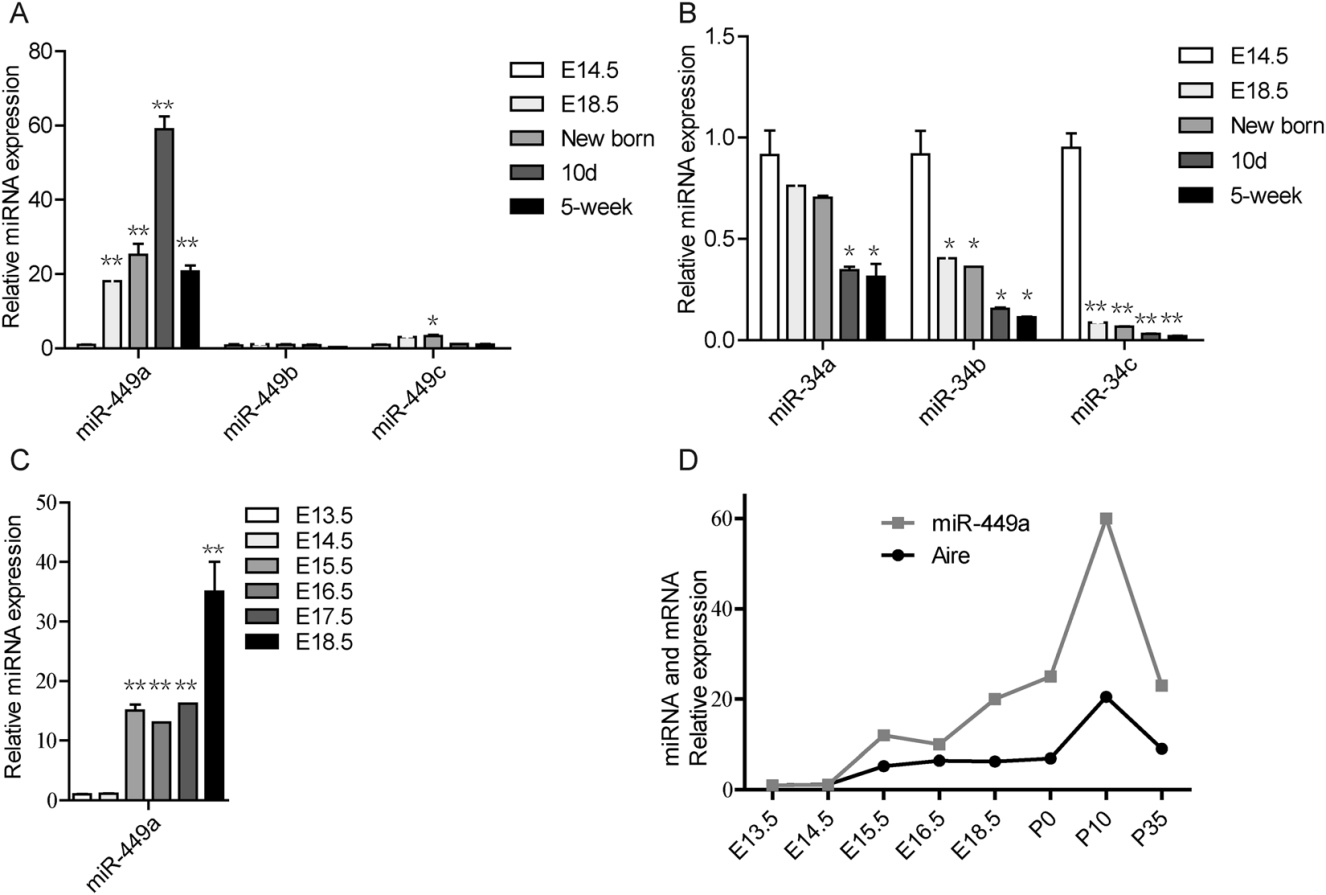
miR-449a expression profiling during thymus development was positively correlated with that of Aire expression. (**A**) q-PCR analyzed the expression of *miR-449a, miR-449b* and *miR-449c* in thymic epithelial cells of E14.5, E18.5, New-born, 10-day old and 5-week old thymi as indicated. (**B**) q-PCR analyzed the expression of *miR-34a, miR-34b* and *miR-34c* in thymic epithelial cells at indicated stages. (**C**) q-PCR analysis of *miR-449a* expression in thymic epithelial cells at indicated stages. (**D**) Comparison of expression profiling of *miR-449a* with that of *Aire* (Gray line for miR-449a, Black line for Aire). Bar graphs show means ± standard errors of at least three independent measurements. (*p < 0.05, **p < 0.01, n = 3).

We further analyzed the temporal expression profiling of these miRNAs in thymus at development stages from E14.5 to 5-week old mice. Expression of miR-449a was dramatically increased at E18.5 and peaked at postnatal 10-day (Fig. 2A). However, the other two members of miR-449 family, miR-449b and miR-449c, showed very low background expression and only miR-449c showed a slight increase in new-born thymi (Fig. 2A). Unlike miR-449 cluster, expression of miR-34 cluster was consistently decreased from the detecting point E14.5 (Fig. 2B). To delicately trace miR-449a expression, we analyzed its expression in thymic lobes at E13.5, E14.5, E15.5, E16.5, E17.5 and E18.5 days. Q-PCR revealed continuous increase of miR-449a expression and a sharp jump at E15.5 (Fig. 2C). Intriguingly, miR-449a and Aire showed very similar temporal expression profiling during thymus development (Fig. 2D), indicating that miR-449a may function to promote mTEC maturation.

### Overexpression of miR-449a induced TEPC differentiation into mature mTEC *in vitro*

**Figure 3:**
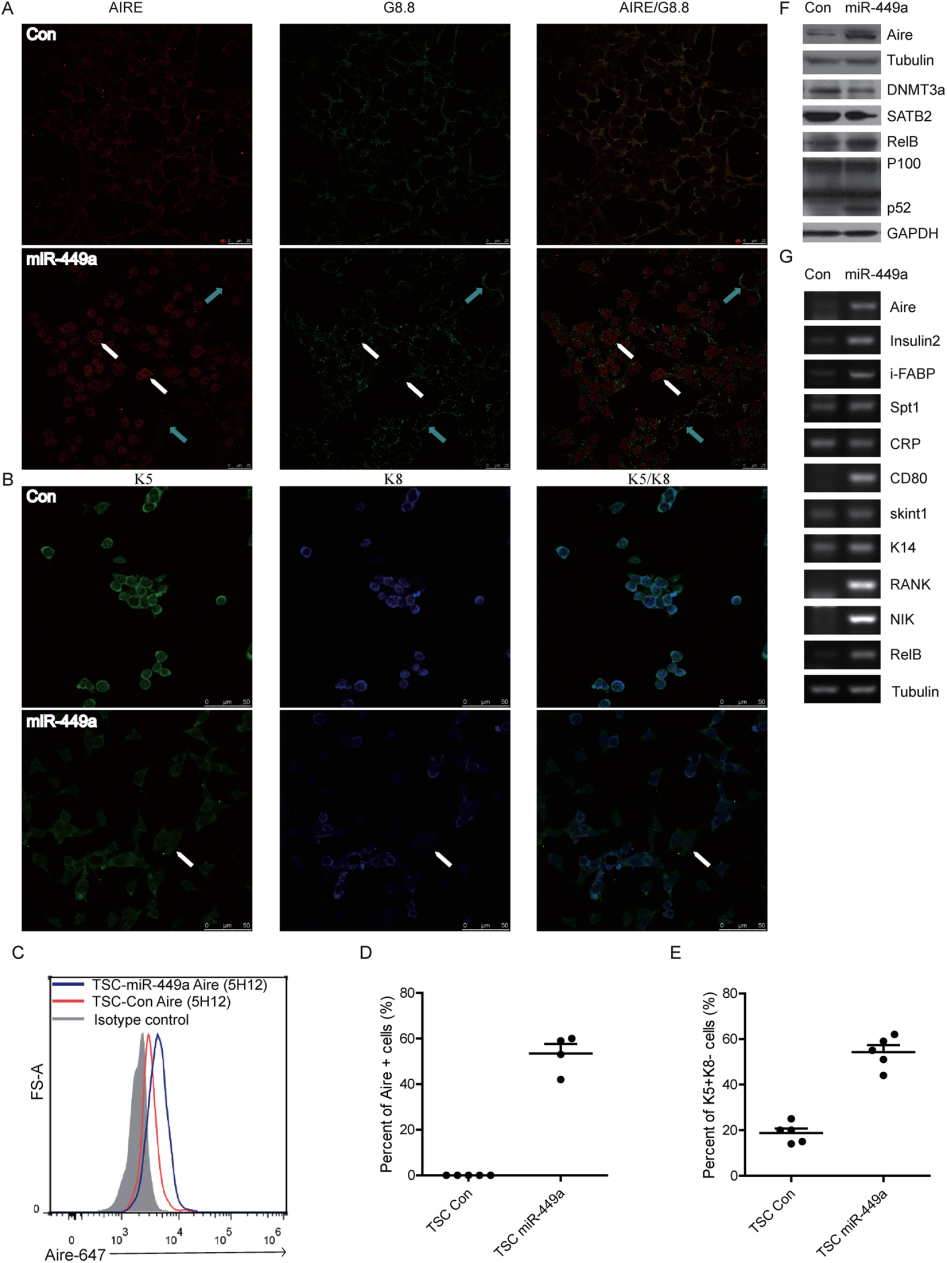
Overexpression of miR-449a induced TEPC differentiation into mature mTEC *in vitro*. (**A**,**B**) Immunofluoresence staining of TSC miR-449a cells and control cells with antibodies: EpCAM (G8.8), Aire (M-300) (**A**), K5 and K8 (**B**). (upper panel: TSC Control, lower panel: TSC miR-449a, white arrow in (**A**): Aire^+^, blue arrow in (**A**): Aire^−^,white arrow in (**B**): K5^+^K8^−^). (**C**) Flow cytometry analysis of TSC miR-449a cells and TSC Control cells with anti-Aire-Alexa 647 (5H12) and isotype control antibody. (**D**) Percent of Aire^+^ TSCs in cultures of TSC miR-449a cells and TSC Control cells from 5 randomly selected view fields of immunofluoresence images in (**A**). (**E**) Percent of K5^+^K8^−^TSCs in cultures of TSC miR-449a cells and TSC Control cells from 5 randomly selected view fields of immunofluoresence images in (**B**). (**F**) Immunoblot analysis of *Aire, RelB, p52, DNMT3a* and *SATB2* in protein extracts of TSC miR-449a cells and TSC Control cells. *GAPDH, Tubulin* act as loading control. (**G**) RT-PCR analysis of indicated genes in TSC miR-449a cells and TSC Control cells, *Tubulin* acts as a loading control. (n ≧ 3).

Aire, a core transcription factor, interacts with a large set of proteins to regulate PTAs expression and is considered as a biomarker of functional mature medullary thymic epithelial cells^19,41^. To see if miR-449a could induce mTEC maturation and Aire expression, we further stably overexpressed miR-449a in TSC cells (labeled as TSC miR-449a cells). Immunofluonrescence staining (Fig. 3A, white arrow: Aire^+^; blue arrow: Aire^−^) and flow cytometry (Fig. 3C) identified about 50% of Aire-expressing TSCs 4 days after miR-449a overexpression. The TEPC population within the fetal thymus has been defined with K5^+^K8^+^ double positive phenotype^42^. Immunofluonrescence staining of TSCs revealed both K5 and K8 expression in control cells while loss of K8 expression in TSC miR-449a cells, thus resulting in K5^+^K8^−^cells reminiscent of K5^+^ mTEC in thymus (Fig. 3B). Counting the Aire^+^ TSC cells in 5 randomly selected view fields from immunofluonrescence images showed about 50% Aire^+^ TSCs in TSC miR-449a cultures while none in TSC control cultures (Fig. 3A and D). There were about 20% K5^+^K8^−^TSCs in TSC control cultures, and this proportion increased to about 55% in TSC miR-449a cultures (Fig. 3B and E).

Western blot assay also detected Aire protein in TSC miR-449a cells (Fig. 3F). At the same time, RelB protein was also increased coupled with the processing of p52, indicating the activation of non-canonical NF-κB during TSC differentiation (Fig. 3F). Interestingly, DNMT3a and SATB2 (2 epigenetic regulators and SATB2 as a miR-449a target in our previous study^43^) were significantly decreased in TSC cells after miR-449a overexpression (Fig. 3F). Further RT-PCR analysis confirmed the thymic identity of TSC miR-449a cells (Fig. 3G).

**Figure 4:**
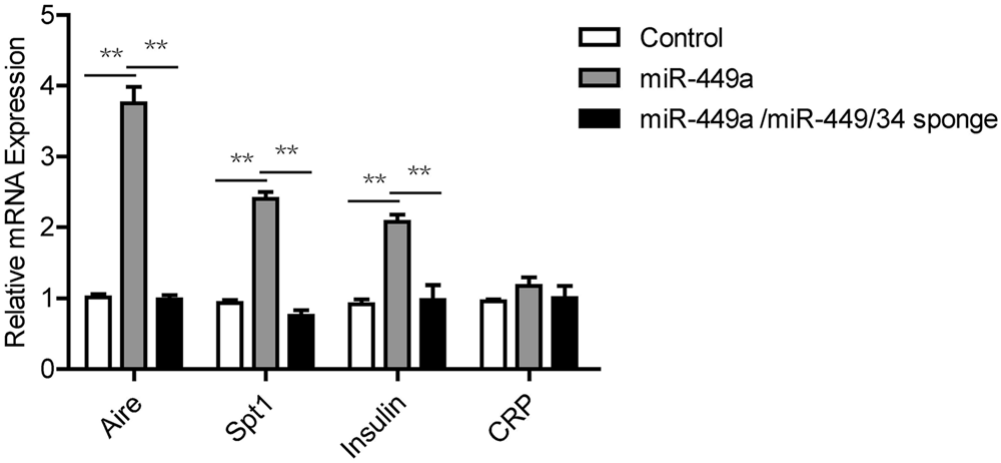
Overexpression of miR-449a induced differentiation of mTEC in 2-DG FTOC. q-PCR analysis of mRNA expression for *aire, spt1, Insulin* and *CRP* in 2-DG FTOC (Control, 2-DG FTOC; miR-449a, 2-DG FTOC infected with lentivirus expressing miR-449a for 4 days; miR-449a/miR-449/34 sponge, 2-DG FTOC infected with lentivirus expressing miR-449a and miR-449/34 sponge for 4 days). Bar graphs show means ± standard errors of at least three independent measurements. (*p < 0.05, **p < 0.01, n = 3).

Furthermore, ectopic overexpression of miR-449a in 2-DG FTOC via lentiviral transduction induced expression of Aire and Aire-dependent PTAs (Fig. 4). Introduction of miR-449/34 sponge that was used to silence miR-449a was sufficient to neutralize the function of miR-449a in 2-DG FTOC (Fig. 4). Taken together, these data demonstrated that in *in vitro* studies using TSC cells or 2-DG FTOC, miR-449a was able to induce mTEC differentiation.

### Mutation of miR-449a alone does not affect thymus development in mouse model

**Figure 5:**
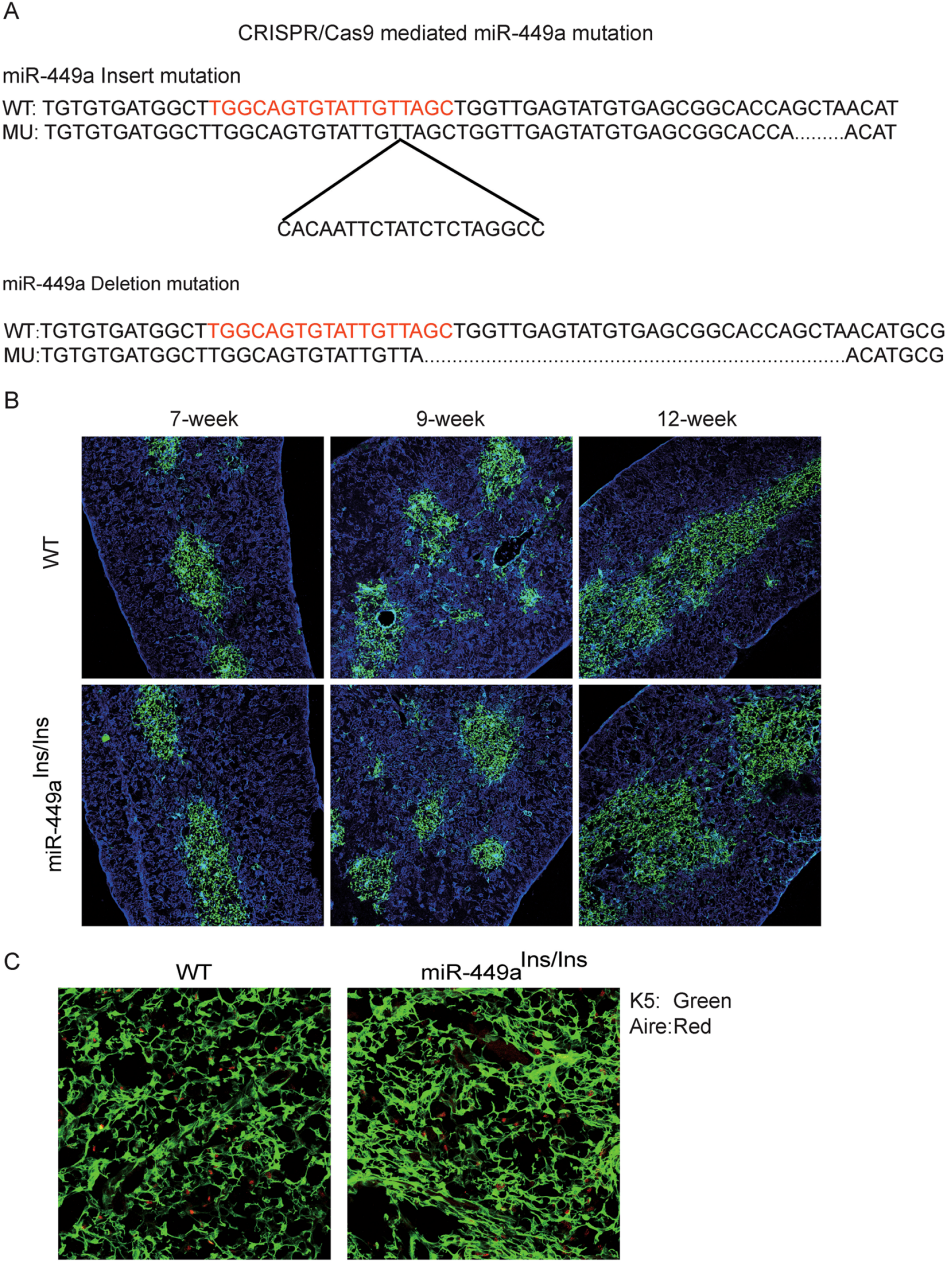
Mutation of miR-449a alone did not affect thymus development in mouse model. (**A**) Sequence information of CRISPR/Cas9 mediated miR-449a mutants. (**B**) Immunofluoresence staining of thymus sections with antibodies to K5 (Green) and K8 (Blue). (**C**) Immunofluoresence staining of 12-week thymus sections with antibodies to K5 (Green) and Aire (Red). (n = 3).

To investigate the function of miR-449a *in vivo*, we generated mouse mutants carrying an insert mutation or a deletion mutation in the miR-449a locus through CRISPR/Cas9 mediated gene editing (Fig. 5A)^44^. The insert mutant strain (miR-449a^Ins/Ins^) bore a 20 bp insertion in miR-449a seed sequence following TGGCAGTGTATTGT while the deletion mutant strain (miR-449a^Del/Del^) bore a 30-bp deletion just after TGGCAGTGTATTGTTA (Fig. 5A). PCR analysis and further DNA sequencing demonstrated that both alleles of miR-449a were mutated in miR-449a^Ins/Ins^ and miR-449a^Del/Del^ mice (Fig. S1A, B). Both mutant strains developed normally without gross defects in any organs and had normal reproductive ability.

We then analyzed the structure of mutant thymus. The mutant thymus displayed the same size as the heterozygous and wild type littermates (Fig. S2). Innunofluorescence staining of thymus sections showed normal thymic medulla and cortex distribution at 7-week, 9-week, and 12-week (Fig. 5B and S3). Aire expression in thymus sections had no significant difference (Fig. 5C). Transcripts for Aire and selected PTAs were quantified in thymic epithelial cells. Consistently, the expression of Aire and PTAs displayed no significant difference between miR-449a mutant mice and wild type littermates (Fig. S4A).

To further confirm if miR-449a was mutated in thymic epithelial cells, we analyzed the mature miR-449a expression in TECs. Q-PCR analysis revealed that mature miR-449a was nearly depleted in miR-449a^Ins/Ins^ (Fig. S4B) and miR-449a^Del/Del^ mice (data not shown). However, the expression of miR-449b was slightly increased(Fig. S4B). The transcripts of miR-449a host gene CDC20b was unaffected indicating that the mutation of miR-449a had no side-effect on host gene expression (Fig. S4C). Collectively, these results suggested that the lack of miR-449a had no overall significant effects on thymus development.

### Expression of miR-34a was increased in miR-449a^Ins/Ins^ thymus

**Figure 6:**
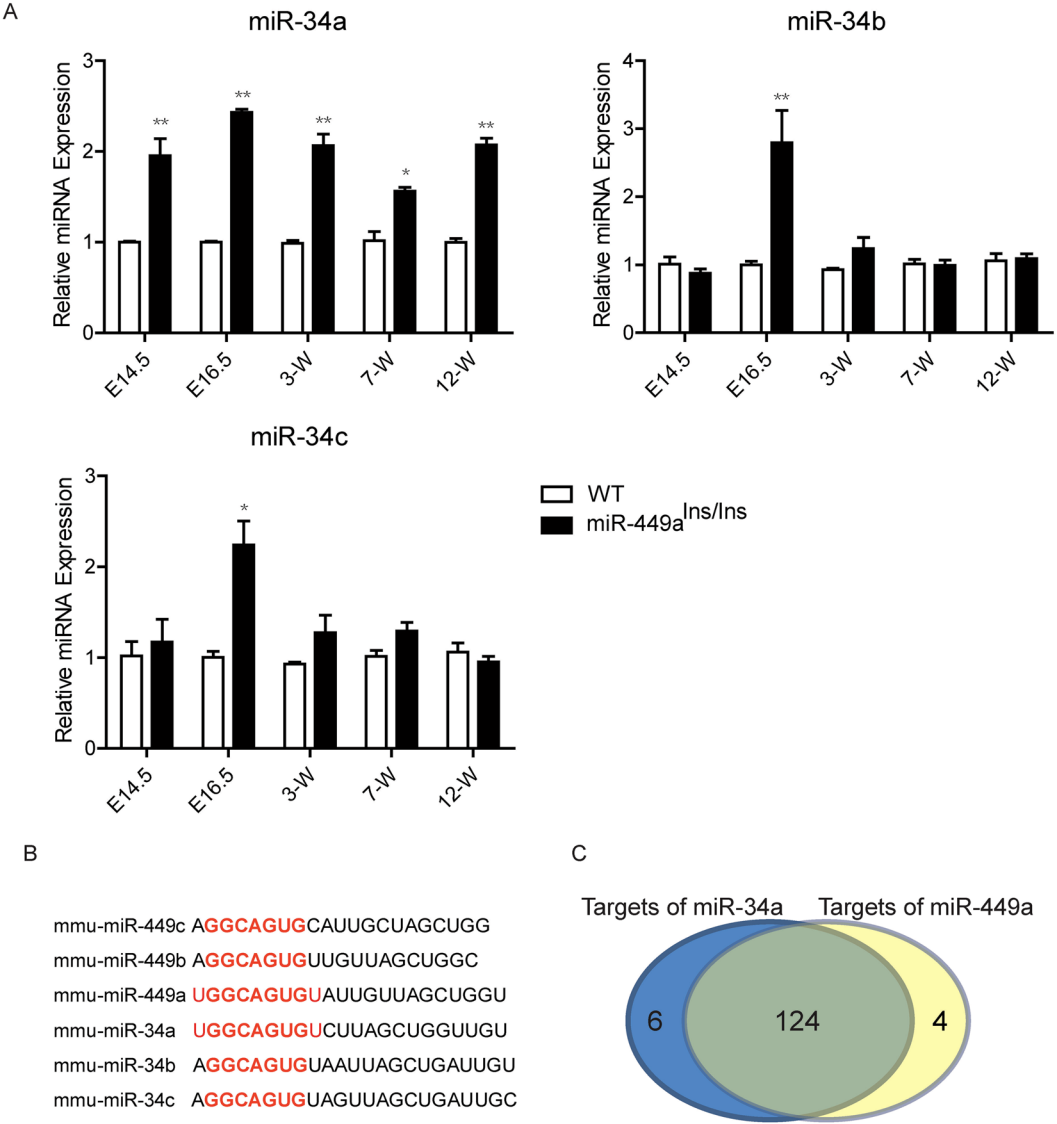
Expression of miR-34a was increased in miR-449a^Ins/Ins^ thymus. (**A**) q-PCR analysis of *miR-34a, miR-34b* and *miR-34c* expression in TECs of E14.5, E16.5, 3-week, 7-week, 12-week old miR-449a^Ins/Ins^ mice and their littermates. (**B**) *In silico* analysis of the miR-449 cluster and miR-34 cluster members. (**C**) 130 candidate targets of miR-34a and 128 candidate targets of miR-449a with target score >85 were predicted by miRDB, containing 124 overlapping candidates. Bar graphs show means ± standard errors of at least three independent measurements. (*p < 0.05, **p < 0.01, n = 3).

The functional redundancy of miR-449/34 family has been previously reported^38–40^. We wondered if this functional redundancy was responsible for the lack of effects of miR-449a mutation on thymus development. We then analyzed the expression of miR-34 cluster members in TECs during thymus development at E14.5, E16.5, 3-week, 7-week and 12-week. To our surprise, miR-34a exhibited a marked increase in miR-449a deficient thymus (Fig. 6A). Mir-34b and miR-34c also showed an increase in E16.5 thymus but not in postnatal thymus (Fig. 6A).

*In silico* analysis identified that members of the miR-449 cluster and miR-34 cluster possess similar mature sequences and seed regions (Fig. 6B). In addition, we analyzed the candidate targets of miR-34a and miR-449a by an online database miRDB^45^. Among the candidate targets with target score >85, most of the targets of miR-34a overlapped that of miR-449a (Fig. 6C and Table 1). Thus, these results supported the notion that the increased expression of miR-34a compensated for the absence of miR-449a in miR-449a-mutant mice.

**Table 1:** List of candidate targets of miR-34a and miR-449a predicted by miRDB.

Predicted targets for mmu-miR-34a in miRDB			Predicted targets for mmu-miR-449a in miRDB		
Target Score	Gene Symbol	Gene Description	Target Score	Gene Symbol	Gene Description
100	Arhgap26	Rho GTPase activating protein 26	100	Erc1	ELKS/RAB6-interacting/CAST family member 1
100	Erc1	ELKS/RAB6-interacting/CAST family member 1	100	Arhgap26	Rho GTPase activating protein 26
100	Vamp2	vesicle-associated membrane protein 2	100	Vamp2	vesicle-associated membrane protein 2
100	Syt1	synaptotagmin I	100	Syt1	synaptotagmin I
99	E2f5	E2F transcription factor 5	99	E2f5	E2F transcription factor 5
99	Tbc1d2b	TBC1 domain family, member 2B	99	Fam76a	family with sequence similarity 76, member A
99	Fam76a	family with sequence similarity 76, member A	99	Tbc1d2b	TBC1 domain family, member 2B
99	Ppp2r5a	protein phosphatase 2, regulatory subunit B′, alpha	99	Mpp2	membrane protein, palmitoylated 2 (MAGUK p55 subfamily member 2)
99	Mpp2	membrane protein, palmitoylated 2 (MAGUK p55 subfamily member 2)	99	Ppp2r5a	protein phosphatase 2, regulatory subunit B′, alpha
99	Ppp1r11	protein phosphatase 1, regulatory (inhibitor) subunit 11	99	Notch1	notch 1
99	Notch1	notch 1	98	Ttc19	tetratricopeptide repeat domain 19
98	Ttc19	tetratricopeptide repeat domain 19	98	Osgin2	oxidative stress induced growth inhibitor family member 2
98	Zfhx4	zinc finger homeodomain 4	98	Zfhx4	zinc finger homeodomain 4
98	Akap6	A kinase (PRKA) anchor protein 6	98	Slc6a1	solute carrier family 6 (neurotransmitter transporter, GABA), member 1
98	Abr	active BCR-related gene	98	Zfp644	zinc finger protein 644
98	Camta1	calmodulin binding transcription activator 1	98	Camta1	calmodulin binding transcription activator 1
98	Slc6a1	solute carrier family 6 (neurotransmitter transporter, GABA), member 1	98	Abr	active BCR-related gene
98	Osgin2	oxidative stress induced growth inhibitor family member 2	98	Slc44a2	solute carrier family 44, member 2
98	Zfp644	zinc finger protein 644	98	Akap6	A kinase (PRKA) anchor protein 6
97	Ubl4	ubiquitin-like 4	97	Mmp25	matrix metallopeptidase 25
97	Hspb6	heat shock protein, alpha-crystallin-related, B6	97	Il6ra	interleukin 6 receptor, alpha
97	Pitpnc1	phosphatidylinositol transfer protein, cytoplasmic 1	97	Mycn	v-myc myelocytomatosis viral related oncogene, neuroblastoma derived (avian)
97	Il6ra	interleukin 6 receptor, alpha	97	Calcr	calcitonin receptor
97	Mmp25	matrix metallopeptidase 25	97	Hspb6	heat shock protein, alpha-crystallin-related, B6
97	Pnoc	prepronociceptin	97	Ppp1r11	protein phosphatase 1, regulatory (inhibitor) subunit 11
97	Mycn	v-myc myelocytomatosis viral related oncogene, neuroblastoma derived (avian)	97	Pitpnc1	phosphatidylinositol transfer protein, cytoplasmic 1
97	Tmem79	transmembrane protein 79	97	Cbfa2t3	core-binding factor, runt domain, alpha subunit 2, translocated to, 3 (human)
97	Zfp775	zinc finger protein 775	97	4930544G11Rik	RIKEN cDNA 4930544G11 gene
97	Cbfa2t3	core-binding factor, runt domain, alpha subunit 2, translocated to, 3 (human)	97	Ubl4	ubiquitin-like 4
97	Soga1	suppressor of glucose, autophagy associated 1	97	Pnoc	prepronociceptin
97	Fbxo30	F-box protein 30	97	Tmem79	transmembrane protein 79
97	Nfam1	Nfat activating molecule with ITAM motif 1	97	Arhgap1	Rho GTPase activating protein 1
97	Calcr	calcitonin receptor	97	Fbxo30	F-box protein 30
97	Arhgap1	Rho GTPase activating protein 1	96	Cntn2	contactin 2
96	Ddx17	DEAD (Asp-Glu-Ala-Asp) box polypeptide 17	96	Ddx17	DEAD (Asp-Glu-Ala-Asp) box polypeptide 17
96	Ctnnd2	catenin (cadherin associated protein), delta 2	96	Mllt3	myeloid/lymphoid or mixed-lineage leukemia (trithorax homolog, Drosophila); translocated to, 3
96	Hexa	hexosaminidase A	96	Ctnnd2	catenin (cadherin associated protein), delta 2
96	Pacs1	phosphofurin acidic cluster sorting protein 1	96	Pacs1	phosphofurin acidic cluster sorting protein 1
96	Cuedc1	CUE domain containing 1	96	Hexa	hexosaminidase A
96	Mllt3	myeloid/lymphoid or mixed-lineage leukemia (trithorax homolog, Drosophila); translocated to, 3	95	Pogz	pogo transposable element with ZNF domain
96	Cntn2	contactin 2	95	Ppp2r3a	protein phosphatase 2, regulatory subunit B″, alpha
95	Pogz	pogo transposable element with ZNF domain	95	Sfmbt2	Scm-like with four mbt domains 2
95	Snx15	sorting nexin 15	95	Dgkz	diacylglycerol kinase zeta
95	Tbl1xr1	transducin (beta)-like 1X-linked receptor 1	95	Tbl1xr1	transducin (beta)-like 1X-linked receptor 1
95	Dgkz	diacylglycerol kinase zeta	95	Lef1	lymphoid enhancer binding factor 1
95	Lef1	lymphoid enhancer binding factor 1	95	Satb2	special AT-rich sequence binding protein 2
95	Sfmbt2	Scm-like with four mbt domains 2	94	Astn1	astrotactin 1
94	Ahcyl2	S-adenosylhomocysteine hydrolase-like 2	94	Eml5	echinoderm microtubule associated protein like 5
94	Slc44a2	solute carrier family 44, member 2	94	Ranbp10	RAN binding protein 10
94	Eml5	echinoderm microtubule associated protein like 5	94	Met	met proto-oncogene
94	Ranbp10	RAN binding protein 10	94	Strn3	striatin, calmodulin binding protein 3
94	Astn1	astrotactin 1	94	Zmym4	zinc finger, MYM-type 4
94	Strn3	striatin, calmodulin binding protein 3	94	Hnf4a	hepatic nuclear factor 4, alpha
94	Met	met proto-oncogene	94	Rfx3	regulatory factor X, 3 (influences HLA class II expression)
94	Hnf4a	hepatic nuclear factor 4, alpha	94	Ahcyl2	S-adenosylhomocysteine hydrolase-like 2
94	Rfx3	regulatory factor X, 3 (influences HLA class II expression)	93	Pkp4	plakophilin 4
94	Zmym4	zinc finger, MYM-type 4	93	Ubp1	upstream binding protein 1
93	Slc4a7	solute carrier family 4, sodium bicarbonate cotransporter, member 7	93	Tmem255a	transmembrane protein 255 A
93	Tmem255a	transmembrane protein 255 A	93	Etl4	enhancer trap locus 4
93	Pkp4	plakophilin 4	93	Slc4a7	solute carrier family 4, sodium bicarbonate cotransporter, member 7
93	Tmem55a	transmembrane protein 55 A	92	Iqgap3	IQ motif containing GTPase activating protein 3
93	Ubp1	upstream binding protein 1	92	Nav3	neuron navigator 3
93	Etl4	enhancer trap locus 4	92	Trim67	tripartite motif-containing 67
92	Scml2	sex comb on midleg-like 2 (Drosophila)	92	Scml2	sex comb on midleg-like 2 (Drosophila)
92	Taf5	TAF5 RNA polymerase II, TATA box binding protein (TBP)-associated factor	92	Papss2	3′-phosphoadenosine 5′-phosphosulfate synthase 2
92	Iqgap3	IQ motif containing GTPase activating protein 3	92	Taf5	TAF5 RNA polymerase II, TATA box binding protein (TBP)-associated factor
92	Uhrf2	ubiquitin-like, containing PHD and RING finger domains 2	92	Plcb1	phospholipase C, beta 1
92	Rtn4rl1	reticulon 4 receptor-like 1	92	Daam1	dishevelled associated activator of morphogenesis 1
92	Daam1	dishevelled associated activator of morphogenesis 1	92	Nat8l	N-acetyltransferase 8-like
92	Papss2	3′-phosphoadenosine 5′-phosphosulfate synthase 2	92	Rtn4rl1	reticulon 4 receptor-like 1
92	Plcb1	phospholipase C, beta 1	92	Uhrf2	ubiquitin-like, containing PHD and RING finger domains 2
92	Trim67	tripartite motif-containing 67	92	Vdr	vitamin D receptor
92	Vdr	vitamin D receptor	92	Slc35g2	solute carrier family 35, member G2
92	4930544G11Rik	RIKEN cDNA 4930544G11 gene	91	Coro1c	coronin, actin binding protein 1 C
92	Nav3	neuron navigator 3	91	Stag3	stromal antigen 3
92	Nat8l	N-acetyltransferase 8-like	91	H6pd	hexose-6-phosphate dehydrogenase (glucose 1-dehydrogenase)
91	Lzts3	leucine zipper, putative tumor suppressor family member 3	91	Crkl	v-crk sarcoma virus CT10 oncogene homolog (avian)-like
91	Stag3	stromal antigen 3	91	Soga1	suppressor of glucose, autophagy associated 1
91	Pea15a	phosphoprotein enriched in astrocytes 15 A	91	Vat1	vesicle amine transport protein 1 homolog (T californica)
91	Padi2	peptidyl arginine deiminase, type II	91	Padi2	peptidyl arginine deiminase, type II
91	H6pd	hexose-6-phosphate dehydrogenase (glucose 1-dehydrogenase)	91	Pea15a	phosphoprotein enriched in astrocytes 15 A
91	Coro1c	coronin, actin binding protein 1 C	91	Rps6ka4	ribosomal protein S6 kinase, polypeptide 4
91	Rps6ka4	ribosomal protein S6 kinase, polypeptide 4	91	Frk	fyn-related kinase
91	Frk	fyn-related kinase	90	Tanc2	tetratricopeptide repeat, ankyrin repeat and coiled-coil containing 2
91	Vat1	vesicle amine transport protein 1 homolog (T californica)	90	Ing5	inhibitor of growth family, member 5
91	Crkl	v-crk sarcoma virus CT10 oncogene homolog (avian)-like	90	Tmem25	transmembrane protein 25
90	Itsn1	intersectin 1 (SH3 domain protein 1 A)	90	Itsn1	intersectin 1 (SH3 domain protein 1 A)
90	Tmem25	transmembrane protein 25	90	Car7	carbonic anhydrase 7
90	Ing5	inhibitor of growth family, member 5	90	Ccne2	cyclin E2
90	Gpr158	G protein-coupled receptor 158	90	Gpr158	G protein-coupled receptor 158
90	Tanc2	tetratricopeptide repeat, ankyrin repeat and coiled-coil containing 2	90	Map1a	microtubule-associated protein 1 A
90	Pdgfrb	platelet derived growth factor receptor, beta polypeptide	89	Cuedc1	CUE domain containing 1
90	Car7	carbonic anhydrase 7	89	Zfp120	zinc finger protein 120
90	Ccne2	cyclin E2	89	Tom1	target of myb1 homolog (chicken)
89	Srpr	signal recognition particle receptor (‘docking protein’)	89	Patz1	POZ (BTB) and AT hook containing zinc finger 1
89	Zfp120	zinc finger protein 120	89	Srpr	signal recognition particle receptor (‘docking protein’)
89	Satb2	special AT-rich sequence binding protein 2	89	Vwa5b2	von Willebrand factor A domain containing 5B2
89	Tom1	target of myb1 homolog (chicken)	89	S1pr3	sphingosine-1-phosphate receptor 3
89	Casp2	caspase 2	89	Nfam1	Nfat activating molecule with ITAM motif 1
89	S1pr3	sphingosine-1-phosphate receptor 3	89	Casp2	caspase 2
89	Vwa5b2	von Willebrand factor A domain containing 5B2	88	Scn2b	sodium channel, voltage-gated, type II, beta
89	Patz1	POZ (BTB) and AT hook containing zinc finger 1	88	Sar1a	
88	Sar1a	SAR1 gene homolog A (S. cerevisiae)	88	Nup210	nucleoporin 210
88	Rragc	Ras-related GTP binding C	88	Pdgfra	platelet derived growth factor receptor, alpha polypeptide
88	Scn2b	sodium channel, voltage-gated, type II, beta	88	Nrip3	nuclear receptor interacting protein 3
88	Nup210	nucleoporin 210	88	Ddn	dendrin
88	Pdgfra	platelet derived growth factor receptor, alpha polypeptide	88	Rragc	Ras-related GTP binding C
88	Ddn	dendrin	87	Scamp4	secretory carrier membrane protein 4
88	Nrip3	nuclear receptor interacting protein 3	87	Tgif2	TGFB-induced factor homeobox 2
87	Fam167a	family with sequence similarity 167, member A	87	Ltbp2	latent transforming growth factor beta binding protein 2
87	Fam46a	family with sequence similarity 46, member A	87	Fam46a	family with sequence similarity 46, member A
87	Wasf1	WAS protein family, member 1	87	Wasf1	WAS protein family, member 1
87	Ltbp2	latent transforming growth factor beta binding protein 2	87	Fam167a	family with sequence similarity 167, member A
87	Tgif2	TGFB-induced factor homeobox 2	87	Stk10	serine/threonine kinase 10
87	Scamp4	secretory carrier membrane protein 4	86	Gmfb	glia maturation factor, beta
87	Stk10	serine/threonine kinase 10	86	Metap1	methionyl aminopeptidase 1
86	Metap1	methionyl aminopeptidase 1	86	Fam131b	family with sequence similarity 131, member B
86	Dpysl4	dihydropyrimidinase-like 4	86	Celf3	CUGBP, Elav-like family member 3
86	Gmfb	glia maturation factor, beta	86	Lman1	lectin, mannose-binding, 1
86	Lman1	lectin, mannose-binding, 1	86	Angpt1	angiopoietin 1
86	Ppp2r3a	protein phosphatase 2, regulatory subunit B″, alpha	86	Isg20	interferon-stimulated protein
86	Celf3	CUGBP, Elav-like family member 3	85	Usf1	upstream transcription factor 1
86	Fam131b	family with sequence similarity 131, member B	85	Gpr165	G protein-coupled receptor 165
85	Dip2c	DIP2 disco-interacting protein 2 homolog C (Drosophila)	85	Gmnc	geminin coiled-coil domain containing
85	Gpr165	G protein-coupled receptor 165	85	Synj1	synaptojanin 1
85	Sec61a1	Sec61 alpha 1 subunit (S. cerevisiae)	85	Dip2c	DIP2 disco-interacting protein 2 homolog C (Drosophila)
85	Gmnc	geminin coiled-coil domain containing	85	Rnf4	ring finger protein 4
85	Synj1	synaptojanin 1	85	Sec61a1	Sec61 alpha 1 subunit (S. cerevisiae)
85	Usf1	upstream transcription factor 1			
85	Rnf4	ring finger protein 4			

### Thymic *in situ* expression of miR-449/34 sponge reduced mature mTECs

**Figure 7:**
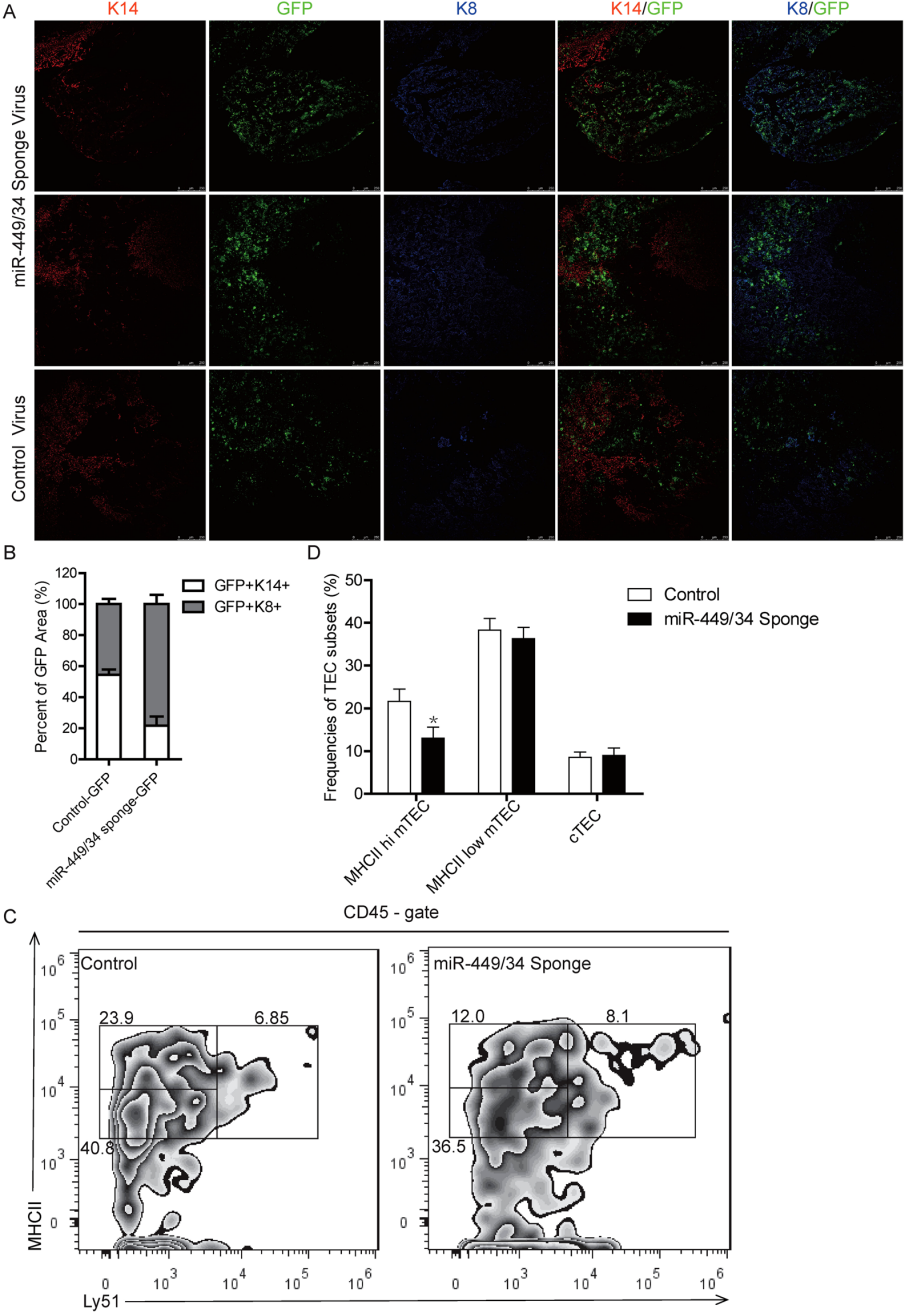
Thymic *in situ* expression of miR-449/34 sponge reduced mature mTECs. (**A**) Immunofluoresence staining of thymus sections after *in situ* injection of control-GFP virus or miR-449/34 sponge-GFP virus with antibodies: K14 (Red) and K8 (Blue), Green indicated GFP expression. (**B**) Statistic analysis of the K14^+^GFP^+^ and K8^+^GFP^+^ zone in GFP-expressing area in 3 immunofluoresence images from (**A**). (**C**) Flow cytometry analysis of TECs after *in situ* injection of control-GFP virus or miR-449/34 sponge-GFP virus with antibodies: CD45-PEcy7, Ly51-Alexa 647, MHCII V500. (**D**) Frequencies of TEC populations (MHCII^hi^ mTEC: MHCII^hi^ Ly51^−^CD45^−^, MHC^low^ mTEC: MHCII^low^ Ly51^−^CD45^−^, cTEC: MHCII^+^ Ly51^+^CD45^−^) in (**C**). Data represent three independent experiments with at least three mice per group. *p < 0.05.

To further confirm the impact of miR-449a on mTEC development, we introduced miR-449/34 sponge-GFP in thymus through *in situ* injection of lentivirus in 3-week old mice. Expression of miR-449/34 sponge resulted in reduced GFP^+^ medulla (K14^+^GFP^+^) and augmented GFP^+^ cortex (K8^+^GFP^+^) in GFP-expression area (Fig. 7A 1^st^ and 2^nd^ panel). However, control-GFP expression could be detected in both medulla and cortex (Fig. 7A 3^rd^ panel). Statistic analysis of the K14^+^ and K8^+^ zone in GFP-expressing area revealed that K14^+^ GFP^+^ mTEC was significantly reduced, while K8^+^ GFP^+^ cTEC was increased after expression of miR-449/34 sponge (Fig. 7B). Flow cytometry analysis of TEC subsets in infected thymus further confirmed reduction of mature MHCII^hi^ mTECs (Fig. 7C and D). Thus, these results indicated that expression of miR-449/34 sponge to interfere with miR-449a and other cluster members blocked normal maturation of mTECs.

## Discussion

The roles of miRNAs in adaptive immune system have been extensively investigated in T cells^46,47^, B cells^48^ and dendritic cells^49^ by conditional dysfunction of RNase III enzyme *Dicer*. Many miRNAs such as miR-146^50^, miR155^51–53^, miR-150^54,55^ have been reported to regulate T cell, B cell and dendritic cell development and function. The deletion of *Dicer* in TECs first revealed the global function of miRNAs in thymus^33,34^. However, the function of a single miRNA that regulates thymic epithelial cell development and function is rarely reported.

Here, analysis of miRNA expression in 2-DG FTOC identified that miR-449a was up-regulated by RANK ligand. Interestingly, by searching for the 3′UTR of mRNA sequence, miR-449a was predicted to target *SATB2*, a transcription factor that was highly expressed in embryonic stem cells^56^. *Satb1* and the closely related *Satb2* proteins regulate gene expression and higher-order chromatin structure of multigene clusters. The expression of *Satb1* and *Satb2* contributes to the plasticity of *Nanog* expression and *SATB2* overexpression may functionally inhibit differentiation^56^. Consistently, expression of *SATB2* was decreased in *in vitro* differentiation of TEPC into mature thymic epithelial cells by miR-449a from our data.

MiR-449a was highly expressed during thymus development and positively correlated with Aire expression. Furthermore, we demonstrated that miR-449a induced thymic epithelial progenitor cells differentiation into mature thymic epithelial cells *in vitro*. Despite the fact that mice with miR-449a mutation showed no discernible phenotype, the role of miR-449a in thymus development could not be entirely excluded because our data suggested that miR-34a compensated for the dysfunction of miR-449a. Although the expression levels of miR-449b, miR-449c, miR-34b and miR-34c were much lower than that of miR-449a, the expression abundance of miR-34a was comparable to that of miR-449a in developing thymus (data not shown). MiR-34a and miR-449a possessed similar seed sequence and had overlapped candidate targets as predicted. More importantly, expression of miR-34a was significantly up-regulated in miR-449a-mutant thymus, which in another way may indicate its compensation role.

To further confirm the impact of miR-449a on mTEC development, we introduced miR-449/34 sponge through thymic *in situ* injection of lentivirus in 3-week old mice. Injection of miR-449/34 sponge virus resulted in reduced GFP^+^ medulla and augmented GFP^+^ cortex, reflected on the extensive miR-449/34 sponge-GFP expression in cortex. Flow cytometry analysis of TEC subsets in infected thymus also showed reduction of mature MHCII^hi^ mTECs. Taken together, these results indicated that interference of miR-449a and miR-449/34 cluster blocked normal differentiation of mTEC and may also have impact on cTEC development. Clues from the expression profiling of miR-449/34 cluster during thymus development, miR-34 may function at early stage before E15.5 while miR-449 may regulate late differentiation of mTECs. The generation of miR-449/34 sponge transgenic mice or miR-449/34 knock out mice may help to reveal the molecular mechanisms of miR-449/34 in regulation of thymus development.

## Electronic supplementary material

Supplementary Information
