# Supplementary material for: microRNA-449a modulates medullary thymic epithelial cell differentiation
Source: Sci Rep. 2017 Nov 21;7:15915. doi: 10.1038/s41598-017-16162-2 (PMC5698406; doi:10.1038/s41598-017-16162-2)

microRNA-449a modulates medullary thymic epithelial cell differentiation

Pengfei Chen[2*], Haohao Zhang[1*], Xiaohua Sun[1], Yiming Hu[1], Wenxia Jiang[1], Zhanjie Liu[1], Sanhong Liu[1] and Xiaoren Zhang[1#]

[1]Key Laboratory of Stem Cell Biology, Institute of Health Sciences, Shanghai Institutes for Biological Sciences, Chinese Academy of Sciences and Shanghai Jiao Tong University School of Medicine, Shanghai, 200031, China

[2]Department of traumatic orthopedics, Shenzhen Longhua District Central Hospital, Shenzhen, 518110, China

[*] These authors contributed equally to this work.

[#] Correspondence should be addressed to: Dr. Xiaoren Zhang (xrzhang@sibs.ac.cn)

Supplements:

Figure S1 Mice genotyping.

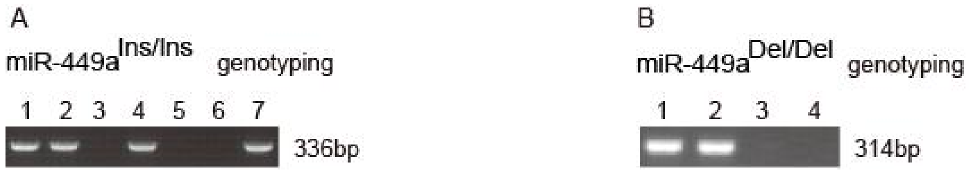

(A) miR-449a$^{Ins/Ins}$ mouse genotyping using primer F1 and R. (No. 3, 5, 6 are wild type; No. 1, 2, 4, 7 are heterozygous or homozygous mutant that need sequencing).
(B) miR-449a$^{Del/Del}$ mouse genotyping using primer F2 and R. (No. 3, 4 are homozygous mutant, further confirmed by sequencing; No. 1, 2 are wild type or heterozygous mutant that need sequencing)

Figure S2 A picture of dissected thymuses from 3-week old miR-449a$^{Ins/Ins}$ and control littermates.

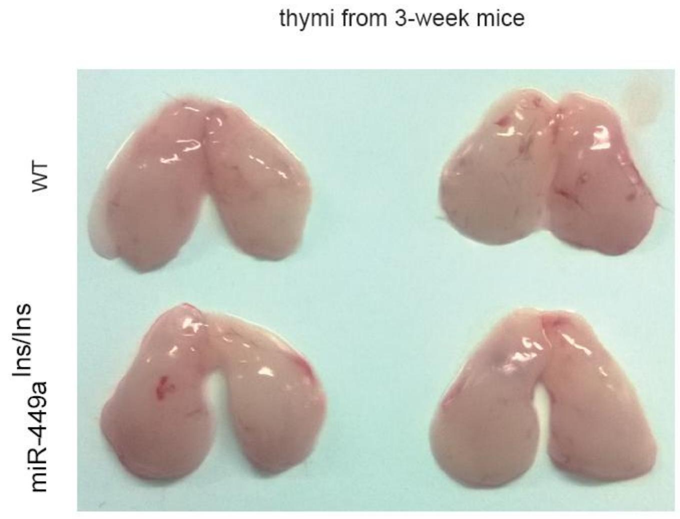

Figure S3 Immunofluoresence staining of thymus sections from 7-week, 9-week and 12-week old mice with antibodies to K5 (Green).

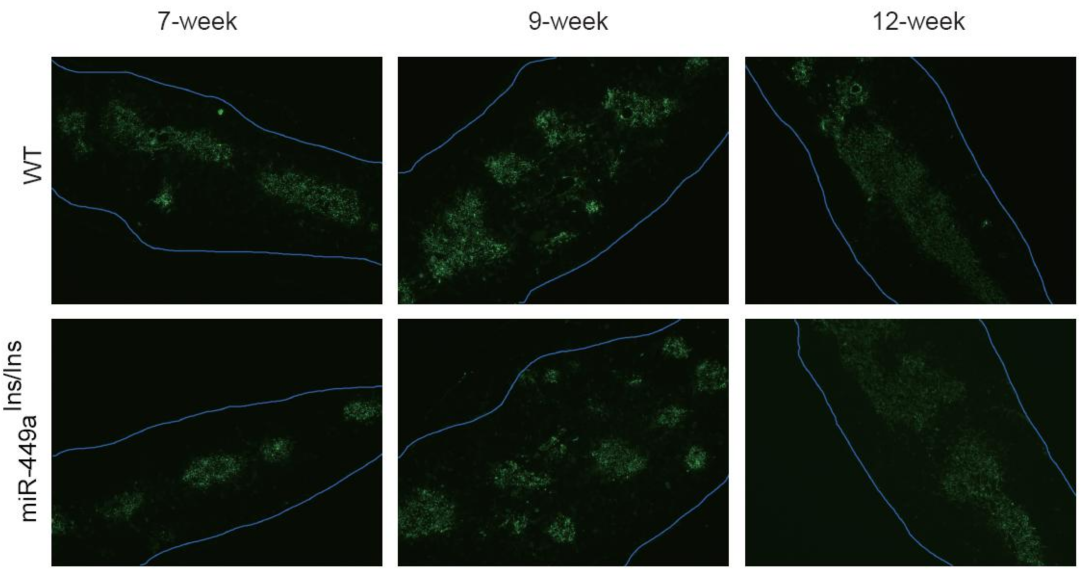

Figure S4 Gene expression in thymic epithelial cells of miR-449a$^{Ins/Ins}$ mice.

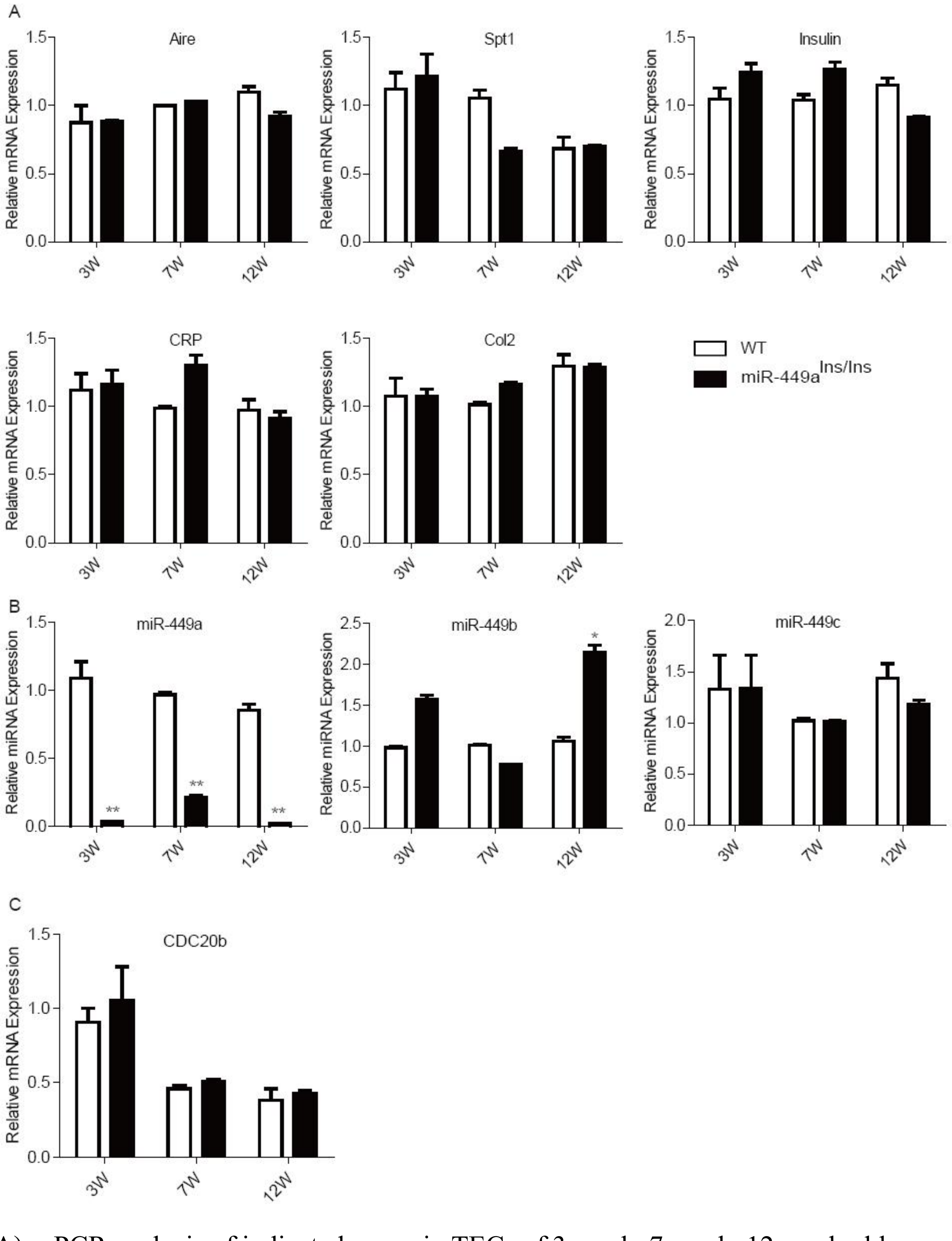

(A) q-PCR analysis of indicated genes in TECs of 3-week, 7-week, 12-week old miR-449a$^{Ins/Ins}$ mice and their littermates.
(B) q-PCR analysis of miR-449a, miR-449b and miR-449c in TECs of 3-week, 7-week, 12-week old miR-449a$^{Ins/Ins}$ mice and their littermates.
(C) q-PCR analysis of CDC20b in TECs of 3-week, 7-week, 12-week old miR-449a$^{Ins/Ins}$ mice and their littermates.
Bar graphs show means±standard erros of at least three independent measurements. (* $p < 0.05$, ** $p<0.01$, $n=3$)

Table 2 List of primers

miR-449a RT	GTCGTATCCAGTGCAGGGTCCGAGGTATTCGCACTGGATACGACACCAGC
miR-449a F	CGGCGGTTGGCAGTGTATTGTTA
miR-449b RT	GTCGTATCCAGTGCAGGGTCCGAGGTATTCGCACTGGATACGACGCCAGC
miR-449b F	CGGCGGTAGGCAGTGTTGTTA
miR-449c RT	GTCGTATCCAGTGCAGGGTCCGAGGTATTCGCACTGGATACGACCCAGCT
miR-449c F	CGGCGGTAGGCAGTGCATTGCT
miR-34a RT	GTCGTATCCAGTGCAGGGTCCGAGGTATTCGCACTGGATACGACACAACC
miR-34a F	CGGCGGTTGGCAGTGTCTTAGCT
miR-34b RT	GTCGTATCCAGTGCAGGGTCCGAGGTATTCGCACTGGATACGACACAATC
miR-34b F	CGGCGGTAGGCAGTGTAATTAGCT
miR-34c RT	GTCGTATCCAGTGCAGGGTCCGAGGTATTCGCACTGGATACGACGCAATC
miR-34c F	CGGCGGTAGGCAGTGTAGTTAGCT
miR-146a RT	GTCGTATCCAGTGCAGGGTCCGAGGTATTCGCACTGGATACGACAACCCA
miR-146a/b F	CGGCGGTTGAGAACTGAATTCCA
miR-146b RT	GTCGTATCCAGTGCAGGGTCCGAGGTATTCGCACTGGATACGACAGCCTA
miR-10a RT	GTCGTATCCAGTGCAGGGTCCGAGGTATTCGCACTGGATACGACCACAAA
miR-10a F	CGGCGGTTACCCTGTAGATCCGAA
miR-132 RT	GTCGTATCCAGTGCAGGGTCCGAGGTATTCGCACTGGATACGACCGACCA
miR-132 F	CGGCGGTTAACAGTCTACAGCCA
miR-192 RT	GTCGTATCCAGTGCAGGGTCCGAGGTATTCGCACTGGATACGACGGCTGT
miR-192 F	CGGCGGTCTGACCTATGAATTG
miR-672 RT	GTCGTATCCAGTGCAGGGTCCGAGGTATTCGCACTGGATACGACTCACAC
miR-672 F	CGGCGGTTGAGGTTGGTGTACTGT
U6 RT	GTCGTATCCAGTGCAGGGTCCGAGGTATTCGCACTGGATACGACAAAATATGGAAC
U6 F	TGCGGGTGCTCGCTTCGGCAGC
miR R Universal	CCAGTGCAGGGTCCGAGGTAT
Aire F	AGGTCAGCTTCAGAGAAAACCA
Aire R	TCATTCCCAGCACTCAGTAGA
spt1 F	CAACACTGAAACGGAAGAAGA
spt1 R	AGCAATGAGAGAGAGGGAGAATAG
Insulin F	AGCCCTAAGTGATCCGCTAC
Insulin R	GCATCCACAGGGCCATGTTG
Col2 F	AGAACAGCATCGCCTACCTG
Col2 R	CTTGCCCCACTTACCAGTGT
CRP F	ATCCCAGCAGCATCCATAGC
CRP R	ACAGTAAAGGTGTTCAGTGGCTTC
SATB2 F	GCCGTGGGAGGTTTGA
SATB2 R	TTCCGCACCAAGACGA
CDC20B F	TAAGCGTCTGTTGTCATTATGTG

CDC20B R	AGCCTCCTTGTGGTAAAGTGTT
Tubulin F	GATCGGTGCTAAGTTCTGGGA
Tubulin R	AGGGACATACTTGCCACCTGT

Full length blots for Fig. 3F

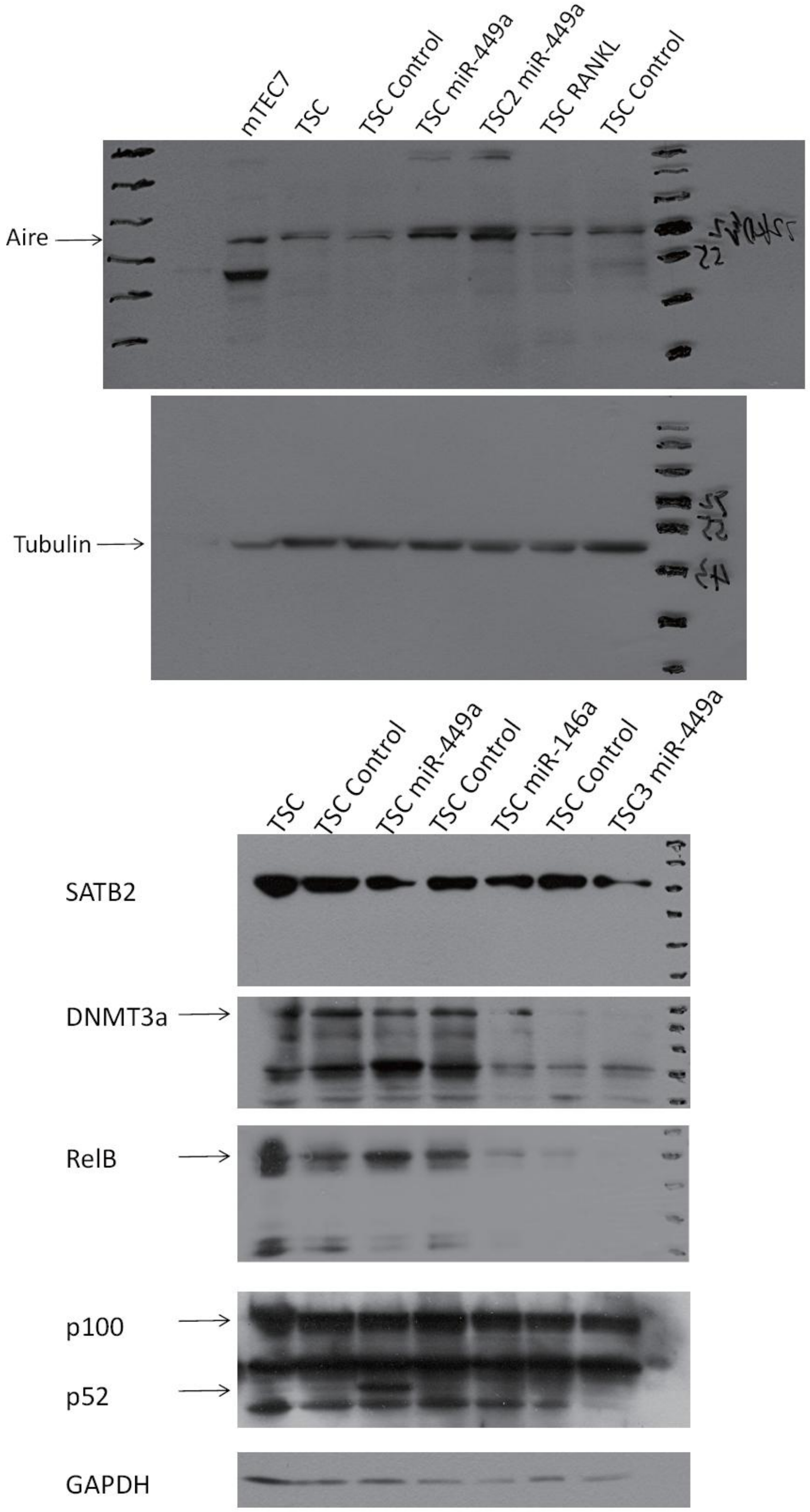

Full length gels for Fig. 3G

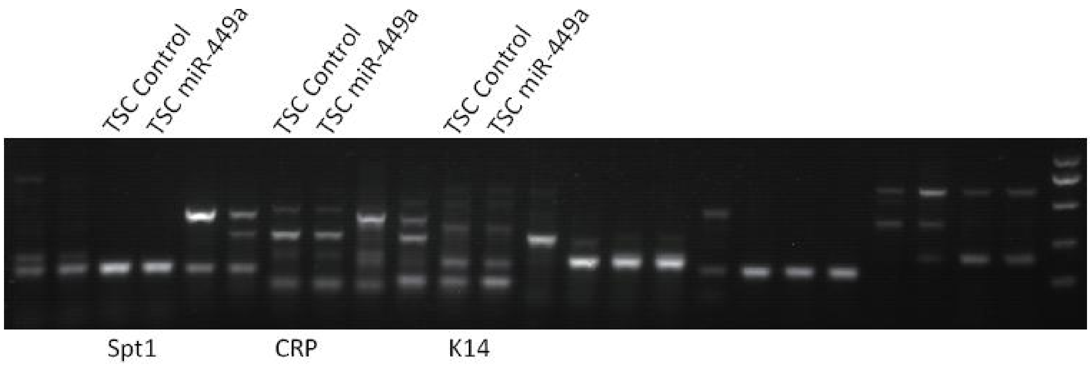

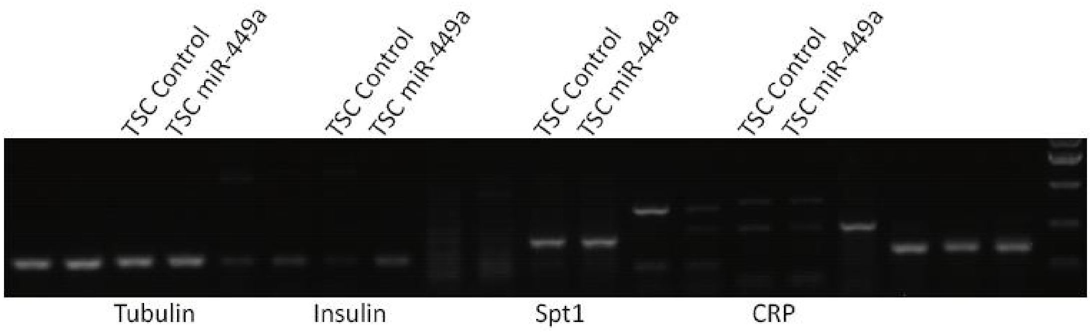

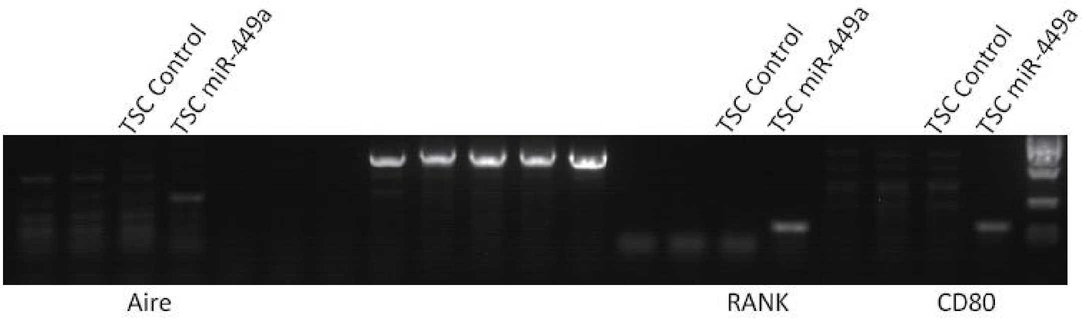

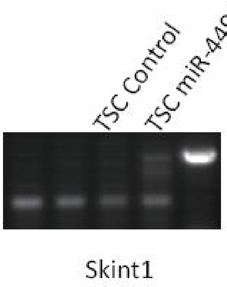

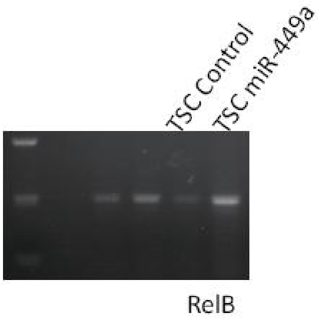

Supplement: Supplementary file 1 — Supplementary Information [file 41598_2017_16162_MOESM1_ESM.pdf]
